# Evaluation of genotoxicity using the micronucleus assay and nuclear abnormalities in the tropical sea fish *Bathygobius soporator* (Valenciennes, 1837) (Teleostei, Gobiidae)

**DOI:** 10.1590/S1415-47572009000200029

**Published:** 2009-06-01

**Authors:** Toni P. Galindo, Lília M. Moreira

**Affiliations:** Departamento de Biologia Geral, Instituto de BiologiaUniversidade Federal da Bahia

**Keywords:** micronucleus assay, nuclear abnormality assay, genotoxicity, frillfin goby, *Bathygobius soporator*

## Abstract

The micronucleus and nuclear abnormalities assays have been used increasingly to evaluate genotoxicity of many compounds in polluted aquatic ecossystems. The aim of this study is to verify the efficiency of the micronucleus assay and nuclear abnormality assay in field and laboratory work, when using erythrocytes of the tropical marine fish *Bathygobius soporator* as genotoxicity biomarkers. Gill peripheral blood samples were obtained from specimens of *Bathygobius soporator*. In order to investigate the frequencies of micronuclei and to assess the sensitivity of species, the results were compared with samples taken at the reference site and maintained in the laboratory, and fish treated with cyclophosphamide. The micronucleus assay was efficient in demonstrating field pollution and reproducing results in the labotatory. There were significant higher frequencies of micronuclei in two sites subject to discharge of urban and industrial effluents. The nuclear abnormality assay did not appear to be an efficient tool for genotoxicity evaluation when compared with field samples taken at a reference site in laboratory, with a positive control.

Most of the world's population has chosen to settle in coastal areas, a choice which has severely impacted these regions in terms of anthropogenic activities, such as urban construction and industrial and domestic effluents. One of the gravest problems of overpopulation, both worldwide and in Brazil, is the lack of efficient sewage-treatment systems.

Historically, the population of the city of Salvador, Bahia State, has always discharged untreated domestic and industrial waste into the coastal area (Souza Santos *et al.*, 2000). In the year 1995, only 26% of the population was benefited by adequate basic sanitary instalations, hence effluent spillings are still common in some places, mainly so during the rainy season.

Studies in the Todos os Santos Bay, where there are some Salvador city beaches, suggested contamination with poisonous and genotoxicant substances such as trace metal, polycyclic aromatic hydrocarbons and polycyclic aliphatic hydrocarbons (Tavares *et al.*, 1988; Hatje *et al.*, 2006). Nowadays, effluents from 29 indusrties drain into the bay, which, together with urban and port ativities, are responsible for considerable pollution problems (Venturini *et al.*, 2004).

Among the various mutagen tests used for bio-monitoring contaminated environments, *e.g.* the comet assay (Buschini *et al.*, 2004), nuclear aberrations (Arkhipchuk and Garanko, 2005), alterations in erythrocytes (Ateeq *et al.*, 2002) and cromosomal aberrations (Ferraro *et al.*, 2004), the micronuclei assay (MN) is relatively simple, reliable and sensitive, and has been used to evaluate the effects of mutagen compounds in many different environments (Al-Sabti and Metcalfe, 1995). Moreover, the micronucleus is composed either of small chromatin fragments which arise as a result of chromosome breaks after clastogenic action, or of whole chromosomes that do not migrate during anaphase as a result of aneugenic affects (Çavas and Ergene-Gözükara, 2003).

Fish have been successfully used in cytogenetic analysis, as they are easy to handle and keep in the laboratory, besides providing a relatively low-cost method (Hayashi *et al.*, 1997). The use of fish erythrocytes allows for quick results with little suffering on the part of the organisms used in bio-monitoring (Minissi *et al.*, 1995). This test enables detecting clastogenic (that break chromosomes) and aneugenic agents (that induce aneuplody or abnormal chromosomal segregation), and has been validated in *in vivo* and *in vitro* experiments (Al-Sabti and Metcalfe, 1995), with a great number of substances being tested in several different organisms, such as mollusks, plants, amphibians, reptiles, birds and mammals (Zúñiga-González *et al.*, 2000; Venier and Zampieron, 2005).

Although several studies have been done with different species of freshwater fish as biomarkers of genotoxicity *in vitro* (Al-Sabti and Metcalfe, 1995; Ateeq *et al.*, 2002) and also in polluted natural environments (Minissi *et al.*, 1995; Hayashi *et al.*, 1997), studies with tropical native species are just beginning. In Brazil there are few laboratory studies that have been done with micronuclei in tropical freshwater fish (Grisolia and Cordeiro, 2000; Grisolia, 2002; Grisolia *et al.*, 2005).

Over recent years, several studies have described the presence of nuclear abnormalities (NA), other than micronuclei, in fish cells exposed to genotoxic substances (Çavas and Ergene-Gözükara, 2005). In general, these abnormalities are considered to be indicators of genotoxic damage, and therefore they may complement micronucleus scoring in routine genotoxicity surveys.

The frillfin goby (*Bathygobius soporator* Valenciennes, 1837) is a small teleost from the Gobiidae family, that lives in tidal pools in coastal regions and oceanic islands on both sides of the Atlantic (Lima *et al.*, 2005). A notable adaptation to the benthic way of life is the development of a sucker formed by uniting the pelvic fins so as to enable it to cling to a substratum (Akihito *et al.*, 2000), and feed on benthic invertebrates, eggs and zooplankton. They live in environments with enormous variations in salinity, oxygen content, turbidity and temperature (Rantin *et al.*, 1998). The species *B. soporator* was selected for the present study, due to its sensitivity, wide distribution and abundance in rocky tidal pools.

The aim of this study is to verify the efficiency of the micronucleus and nuclear abnormalities assays, both in the field and laboratory, with erythrocytes of the tropical marine fish *Bathygobius soporator*, when these are used as biomarkers of genotoxicity.

Ten specimens of *B. soporator* were collected at Stella Mares in January, 2006 and kept in an aquarium (72 L, distilled water and aquarium marine salt), at a constant temperature (30 ± 3 °C) and salinity of 30%o, and fed with commercial fish food for 90 days. After this period, the first blood samples were taken and slides prepared for analysis of erythrocyte frequency, with micronuclei as a negative control. After fifty-four days, the fish were weighed and measured (average weight 7.47 g and length 8.22 cm), and then submitted to an intra-peritoneal injection of cyclophosphamide (Merck) in a saline solution (4%) with a concentration weight of 40 mg/kg. Blood was collected after five days, when slides were prepared and analyzed for positive control.

Ten specimens of *B. soporator* were randomly collected at Stella Mares Beach (12° 57' 05” S; 38° 20' 33” W), Boa Viagem Beach (13° 00' 38” S; 38° 31' 59” W) and Penha Beach (12° 54' 43” S; 38° 29' 38” W), during March, May, August and November, 2005.

Besides the average length of the fish (7.22 cm), temperature, salinity and pH were measured in three replicates. Erythrocyte smears were obtained with heparinized syringes by puncturing the gills on previously washed microscopic slides. The fish remained unharmed and were soon returned to their natural habitat. The slides were air-dried for 24 h, fixed in a 70% methanol solution for 7 min and then dried a further 24 h. Shortly after, they were stained with Giemsa (4%) for 15 min. 3.000 intact erythrocytes were counted from each fish. Only cells that were clearly visible and isolated under a Zeiss microscope with amplification of 1000 X, were counted. Cells with more than four micronuclei were discarded so as to exclude apoptotic phenomena (Bolognesi *et al.*, 2006). Nuclear abnormalities were manifest as changes in the normal elliptic shape of nuclei (Ferraro *et al.*, 2004). For a detailed description on nuclear abnormalities see Ayllón and Garcia-Vazquez (2000), Çavas and Ergene-Gözükara (2003) and Çavas *et al.* (2005). Micronuclei were considered as small inclusions of nuclear material inside erythrocytic cytoplasm. Criteria for identification were a round or oval shape with a flat and well-defined outline, coloration similar to that of the main nucleus and a size from 1/3 to 1/20 in relation to that of the main nucleus (Al-Sabti and Metcalfe, 1995).

Data were tested for normality via the Kolmogorov-Smirnov test and the Bartlett test before all statistical analyses, in order to check variance homogeneity. Micronucleus frequency presented normal distribution and homogeneous variances, whereupon a one way ANOVA was performed, followed by an *a posteriori* Dunnett test. The frequency of nuclear abnormalities did not present homogeneous variances. Neverthless, a non-parametric Kruskall-Wallis test was done, followed by the Dunn multiple comparison test with SPSS 11.0 for Windows. The initial level of significance (α) was 0.1 to compensate for the increased likelihood of a type 2 error, and a Bonferroni correction was applied, this resulting in alfa 0.025.

The results show the average frequencies of micronuclei and nuclear abnormalities in the three sites during four seasons of the year 2005 (Table 1). The frequency of micronuclei was significantly higher than in the negative control at Boa Viagem and Penha in March, May and August. Stella Mares showed a low frequency of miconuclei in comparison to the other two sampling sites, with lower frequencies in November (precipitation of 70 mm), intermediate in the months of May (200 mm) and August (120 mm) and higher in March (350 mm). Boa Viagem presented the highest frequency of miconuclei (Figure 1). There was a significant difference between frequency of miconuclei among the negative and positive controls. However, this pattern was not observed in the frequency of nuclear abnormalities among the two control groups in spite of it being higher in the positive control group. The nuclear abnormalities in the negative control group were higher than those in the other three sites, in all the months, except in Boa Viagem for November (Table 1). Over the sampling period, temperature varied between 23 °C (August in Penha Beach) and 40 °C (March in Stella Mares Beach). The pH varied between 7.49 and 8.94 in all sites. Salinity varied between 14 and 36 %o.

The results of this study confirm the usefulness of the erythrocyte micronucleus as a powerful monitoring tool for detecting genotoxic agents in a coastal environment. Micronuclei frequencies proved to be very reliable for testing genotoxicity in studies *in situ* and *in vitro*, as it was possible to compare results obtained in the field with those from the laboratory. The present study suggests that nuclear abnormalities are not good indicators for genotoxicity evaluation in field studies, since their frequencies were higher in the negative and positive control groups in relation to the sites and months of collection.. It is worth emphasizing that the mechanisms of formation of these nuclear abnormalities are not yet fully understood (Çavas and Ergene-Gözükara, 2003). The results of studies with nuclear abnormalities demonstrate their effectiveness as genotoxicity markers mainly in fresh water fishes and in the laboratory under controlled conditions (Ayllón and Garcia-Vazquez, 2000; Çavas and Ergene-Gözükara, 2005), although it is also necessary to test these parameters in field studies. The need for controls is emphasized in the laboratory for comparison with field studies as a means of reaching more reliable conclusions, since it is through this that the effectiveness of the micronucleus test and not of the nuclear abnormality assay was corroborated. Uncertainties in the extrapolation of laboratory data to natural ecosystems will always exist, as many physical, chemical and biological factors are wholly integrated into the aquatic environment, thereby being very difficult to reproduce. Furthermore, standard laboratory conditions are very different from those in nature (Araújo *et al.*, 2006). Several studies have demonstrated increases in micronuclei frequency in species of marine fish in polluted areas, and their use as genotoxicity markers in accordance with the present study (Al-Sabti and Metcalfe, 1995; Hayashi *et al.*, 1998), and also in the laboratory (Ateeq *et al.*, 2002; Teles *et al.*, 2003; Buschini *et al.*, 2004; Çavas *et al.*, 2005). Other authors have demonstrated the success of using the micronucleus test in the evaluation of environmental quality, by using several freshwater fish (Minissi *et al.*, 1996; Hayashi *et al.*, 1998).

A clear seasonal variation was observed in the frequency of micronuclei and nuclear abnormality. Precipitation seems to be a relevant variable in relation to the increase in micronuclei frequency. Micronuclei frequency in the least rainy month (November, 70 mm) was lower than in the rainiest (March, 350 mm) in all the three reference locations. Nuclear abnormality frequency was the lowest in March and the highest in November in all the sites. In the rainier months, surface-water runoff carries chemical drainage into streams or rivers and finally to beaches. Urban storm-water runoff is now recognized as a major source of pollutants in receiving waters, and a number of recent investigations have traced oil and grease, nutrients (Kayhanian *et al.*, 2007), total hydrocarbons, PHAs and heavy metals (Legret and Pagotto, 1999; Davis *et al.*, 2000) in runoff waters. Additionally, large quantities of industrial and urban effluents are discharged into rivers, ponds and directly into the sea. These effluents might contain organic, inorganic and metallic substances with potential genotoxicants such as heavy metals, PHAs, PCB and pesticides (Claxton *et al.*, 1998; White and Rasmussen, 1998).

From this study, it can be concluded that the micronucleus test as applied to the sea fish *B. soporator* showed to be very efficient in determining genotoxicity in impacted coastal areas. This fish is a sensitive species for bio-monitoring, as well as being an abundant tropical species, easily kept in the laboratory and with a wide distribution along the Brazilian coast. Hence, Sánchez-Galán *et al.* (2001) suggested that benthic fishes are more useful for monitoring sediment contamination. The nuclear abnormality assay did not prove to be a reliable tool for genotoxicity evaluation in field studies, when compared with negative and positive controls. Monthly rainfall constitutes a variable that seems to be associated with micronuclei frequency, seeing that there was a decrease in frequency during the less rainy months and an increase in the months with higher rainfall.

**Figure 1:**
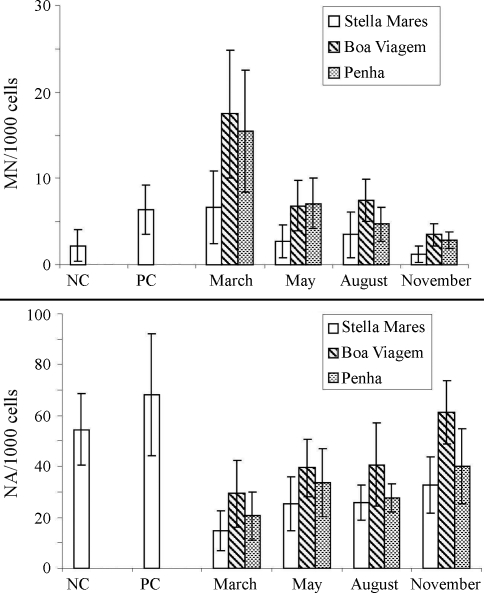
Precipitation during four months of 2005 and mean frequency of micronucleated erythrocytes and nuclear abnormalities in *B. soporator* at three sites in and, Salvador-Bahia, Brazil. NC: negative control; NP: positive control; Prec. mm/months: precipitation mm/months. MN: micronucleus; NA: nuclear abnormalities.

## Figures and Tables

**Table 1:** Average frequency of micronucleus and nuclear abnormalities in *B. soporator* for 1000/cells during four months in 2005 at three sites in Salvador, Bahia, Brazil.

Month	Site	MN ± SD	NA ± SD
Negative control		2.23 ± 1.79	54.50 ± 14.00
Positive control		6.40 ± 2.84**	68.33 ± 24.03

March	Stella Mares	6.63 ± 4.18	14.63 ± 7.82***
	Boa Viagem	17.47 ± 7.43***	29.28 ± 13.06***
	Penha	15.53 ± 7.07***	20.60 ± 9.47**

May	Stella Mares	2.70 ± 1.91	25.37 ± 10.61***
	Boa Viagem	6.83 ± 2.90**	39.50 ± 11.21
	Penha	7.10 ± 2.91***	33.77 ± 13.46*

August	Stella Mares	3.47 ± 2.64	25.80 ± 6.98***
	Boa Viagem	7.43 ± 2.43***	40.73 ± 16.49
	Penha	4.70 ± 2.00	27.80 ± 5.60***

November	Stella Mares	1.20 ± 0.99	32.70 ± 11.00*
	Boa Viagem	3.50 ± 1.28	61.27 ± 12.62
	Penha	2.87 ± 0.94	40.27 ± 14.71

MN: micronucleus; NA: nuclear abnormalities; S.D.: standard deviation. Treatments compared to the negative control by the test of multiple comparisons. (α = 0.025) *Significantly p < 0.025. **Very Significantly p < 0.01 ***Extremely Significantly p < 0.001.
